# Oral Lichen Planus and Polycythemia: Possible Association

**DOI:** 10.1155/2020/8820114

**Published:** 2020-08-05

**Authors:** Yassine Oueslati, Raouaa Belkacem Chebil, Haifa Regaieg, Lamia Oualha, Nabiha Douki

**Affiliations:** ^1^Department of Oral Medicine and Oral Surgery, SAHLOUL Hospital (Sousse), Dental Faculty of Monastir, University of Monastir, Tunisia; ^2^Laboratory of Oral Health and Maxillofacial Rehabilitation (LR12ES11), University of Monastir, Tunisia; ^3^Department of Haematology, Farhat Hached Hospital (Sousse), University of Sousse, Tunisia

## Abstract

Oral lichen planus is a chronic inflammatory disease of established immune-mediated pathogenesis that affects the oral mucosa. Polycythemia is a nonaggressive myeloproliferative disorder, characterized by an increase in red blood cell mass, often with uncontrolled production of granulocytes and platelets. Their association was rarely mentioned in the scientific literature. The aim of this paper was to report their occurrence in a 52-year-old male patient. Although a casual connection cannot be excluded, both diseases share many similarities in the immune dysfunctions involved in their pathogenesis and their clinical features. Such a hypothesis remains to be demonstrated by further studies. The presence of oral lesions should alert the clinicians in the process of identifying and early diagnosing these diseases. Thus, complications can be prevented and treatment can be started at an early stage, avoiding further damage.

## 1. Introduction

Lichen planus was described for the first time in 1869 by Erasmus Wilson as a chronic disease that affects the skin, scalp, nails, and mucosa with possible rare malignant transformation [1]. In fact, the oral variant, oral lichen planus (OLP), is a designated oral potentially malignant disorder (OPMD), showing 1.09% overall rate of malignant transformation [2]. It is a chronic inflammatory disease with characteristic relapses and remissions. Its etiology remains unclear, and the currently accepted pathogenesis is that of a cell-mediated autoimmune disease. The diagnostic criteria for oral lichen planus (OLP) are based on clinical and histopathologic features of the condition according to the World Health Organization classification of 2003 [3]. Its occurrence in association with various systemic diseases does not stop attracting attention.

Polycythaemia was first reported in the medical literature in 1892 [4]. It is a nonaggressive myeloproliferative disorder, characterized by an increase in red blood cell mass, often with uncontrolled production of granulocytes and platelets. Its diagnosis and management is challenging for the medical team. It is reported to affect the oral mucosa [5].

The purpose of this paper was to report the case of a patient with secondary polycythemia who was followed in the Department of “Oral Medicine-Oral Surgery” for oral lichen planus (OLP). We aimed to draw attention to the possible association of these two diseases which has very limited data in the literature and to emphasize the different clinical and histological aspects of the oral mucosal lesions as well as the therapeutic approach followed for the management of the patient.

## 2. Case Report

**Figure 1:**
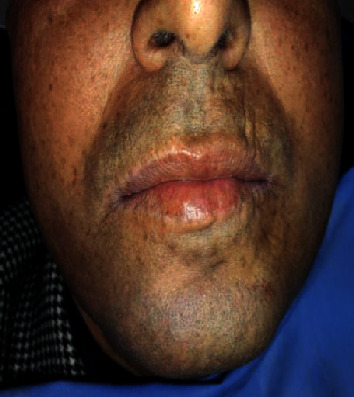
Front view. Slight facial erythrosis.

**Figure 2:**
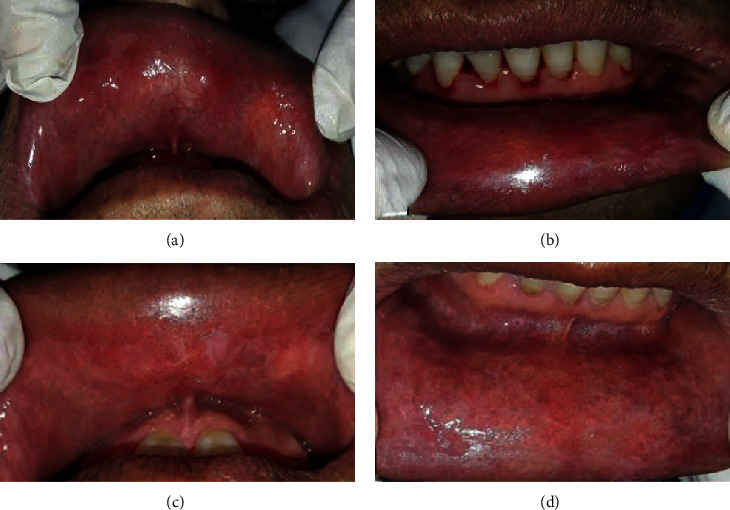
Intraoral view: inner side of the labial mucosa in different stages of the treatment. (a, b) Widespread redness with erosive lesions. (c, d) Disappearance of the erosions with the presence of slight reticular pattern.

**Figure 3:**
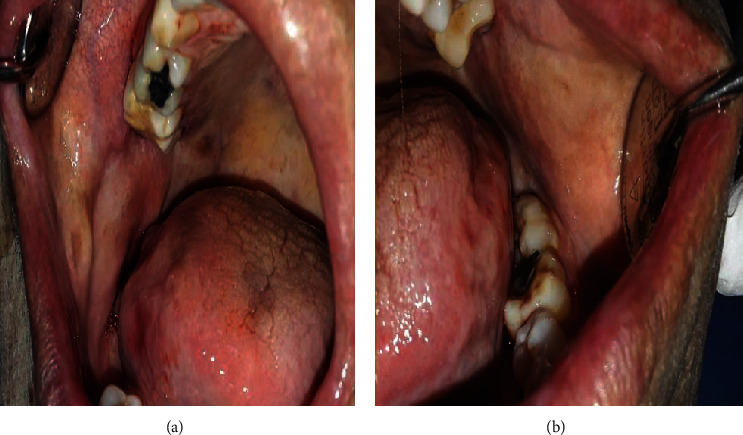
Intraoral view: inner sides of the cheeks. Widespread redness with erythema, erosions, and some pigmented lesions induced by hydroxyurea.

**Figure 4:**
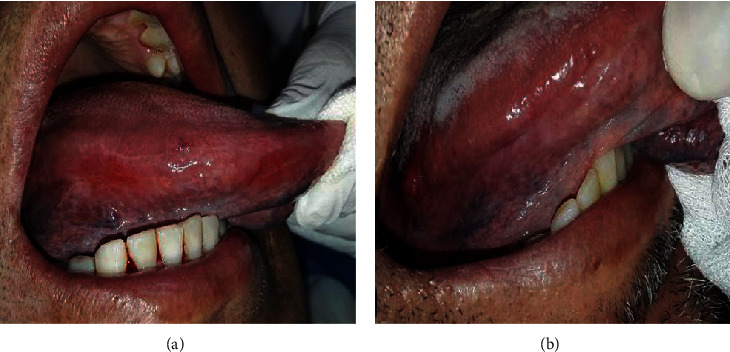
Intraoral view: right lateral edge and ventral side of the tongue in different stages of the treatment. (a) Two painful erosive ulcerations. (b) Improvement of the lesions and disappearance of the ulcerations. We notice the presence of a discrete reticular pattern that characterizes oral lichen planus.

A 52-year-old man with a medical history of high blood pressure and gout was referred to our department for oral mucosa burning causing him pain and discomfort. He was an ancient smoker and reported the sensation of headache with notorious dizziness on two occasions recently. The extraoral examination showed slight facial erythrosis (Figure 1). Intraoral examination showed slightly hyperkeratotic erythematous lesions located on the inner side of the labial mucosa (Figure 2). They were pruriginous and painful causing the symptomatology of the patient. The inner sides of the cheeks showed erythematous erosions especially on the right side (Figure 3). We noticed also two erosive ulcerations as well as hyperkeratotic lesions at the right lateral edge and the ventral side of the tongue forming a discrete reticular network (Figure 4).

**Figure 5:**
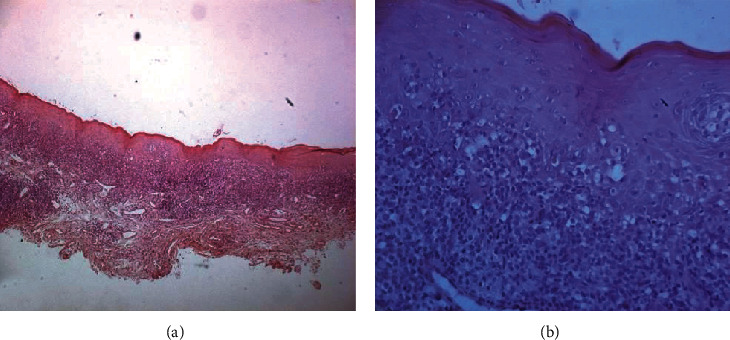
Histological examination leading to the diagnosis of oral lichen planus (OLP). The mucosa was covered with a squamous epithelium taking on a flattened appearance with slight acanthosis and hyperkeratosis (essentially orthokeratosis and some focal parakeratosis). The basement membrane was vacuolated with the presence of Civatte apoptotic bodies (b). The underlying tissue is the seat of a dense linear infiltrate surrounding the nerve nets in places; it is mainly made of lymphocytes with rare cells of a histiocytic nature. This lymphocytic band infiltrate (a) at the level of the chorion attacks and gradually destroys the keratinocytes of the basal and deep seat of the epithelium. No signs of malignancy were noted.

In front of these signs, we suspected oral lichen planus associated with candidiasis infection. Mycological examination confirmed candidiasis, and initial treatment consisted of topical mouthwashes with Amphotericin B (50 mg; 5 ml 3 times per day) and Chlorhexidine (0.12%; 3 times per day). Biological tests were requested and a biopsy was scheduled a week after. Two biopsies were performed at the inner side of the lower lip and the right lateral edge of the tongue. The result was oral lichen planus (Figure 5).

Almost all the biological tests (VS, CRP, glycated hemoglobin, and thyroid checkup) were within the normal levels, and serologies (hepatitis and VIH) were negative. However, the complete blood count was unusually marked by an increase in the number of red blood cells, hematocrit, and hemoglobin reaching a value of 19 (Table 1). This fortuitous finding led us to suspect polycythemia vera and to refer our patient to the department of hematology where he has undergone an emergency phlebotomy. Two days later, he was a victim of a myocardial infraction. He was hospitalized in the cardiology department where he was diagnosed with atrial fibrillation, stented, and put under two platelet aggregation inhibitors: Clopidogrel (Plavix) and Acetylsalicylic acid (Kardégic).

The patient was a candidate for a genetic test looking for the JAK-2(V617F) mutation. The absence of this latter made it possible to rule out the diagnosis of Vasquez's primary polycythemia. Serum erythropoietin (EPO) level was high (109 mIU/mL). Bone marrow biopsy was not realized due to bleeding risk. The final diagnosis was secondary polycythemia due to chronic hypoxia.

The treatment plan was multidisciplinary. In the hematology department, management of polycythemia with hydroxyurea and allopurinol drugs resulted in a durable remission of the myeloproliferative disorder without toxic side effects and a notable regression in the oral pain. There was an appearance of some pigmented oral lesions possibly related to use of hydroxyurea. In conjunction, in the oral medicine department, oral hygiene conditioning, and a prescription of topical corticosteroid (Prednisolone) and Chlorhexidine-based mouthwashes resulted in a clear improvement of the symptomatology of the patient. Actually, he benefits from regular follow-up appointments in our department as well as in the hematology and cardiology departments. His condition is stable. Improvement of biological variables along the treatment is indicated in Table 1.

## 3. Discussion

On the one hand, polycythaemia is a myeloproliferative disorder that refers to an increase in the absolute red blood cell (RBC) mass in the body. In practice, this is reflected by an increase in hemoglobin and hematocrit values over what is considered physiologic for that age and gender [6]. Its prevalence has been estimated to be approximately 22 cases/100000 populations [7].

We distinguish two mechanisms: primary polycythemia known also as “polycythemia vera (PV)” or “Vasquez's disease” and is due to an abnormality of the stem cells of the hematopoietic marrow which acquire tumoural characteristics, proliferate, and cause an overproduction and anomalies of all 3 cell lines, but with a notable prominence of red blood cells [6]. It is usually associated with a gene mutation (V617F) of the JAK 2 (Janus Kinase 2) gene [4]. Secondary polycythemia is linked to an increase in the blood level of erythropoiesis-stimulating hormones, in particular EPO (erythropoietin), most often secondary to chronic hypoxia (hypoxia consists of insufficient oxygenation of the tissues, tissue ischemia causes stimulation of the secretion of EPO by the kidney) [8]. As for our patient, the diagnosis of secondary polycythemia was retained according to the WHO diagnostic criteria for polycythemia vera of 2016 (Table 2) [9].

Secondary polycythaemia is associated primarily with complications arising from hyperviscosity. There is a preponderance of both arterial and venous thrombosis [6] that can sometimes lead to death. As for our patient, he was a victim of a thrombotic event (myocardial infarction) two days after his phlebotomy. He is continuing to follow a cardiology regular monitoring.

Among the oral manifestations of this hematological disorder are erythematous lesions with widespread redness of the mucosa, pruritus, erosions, and ulcerations, things that were seen in the reported case. Petechiae, ecchymosis, and exaggerated varicosities in the ventral tongue are observed in patients with platelet abnormalities [10]. The clinical manifestations include also pale mucosa, reactive keratosis, and different forms of candidiasis [4]. We found candidiasis infection in our patient, and it was successfully managed with antifungal treatment.

On the other hand, lichen planus is a chronic systemic disease of established immune-mediated pathogenesis. The reported prevalence rates of oral lichen planus (OLP) vary from 0.5% to 2.2% of the population [11]. Clinical features of oral lichen planus include multiple mucosal lesions that have a bilateral, symmetrical distribution. The most frequent location is the postero-inferior part of the jugal mucosa, followed by the back of the tongue, the alveolar and vestibular gingival mucosa, more rarely the mucosa and semi-mucosa of the lips, the palate, and the oral floor [12]. In our case, we observed a preponderance of erythematous mucosal lesions associated with pruritus, oral burns, and pain leading to functional discomfort.

The clinical features alone may be sufficiently diagnostic, particularly when presenting in the “classic” reticular form. The need and value of biopsy for histological confirmation of the diagnosis is not definitive [11]. It was indicated in our case because the disease did not present with its typical manifestations and the mucosal lesions were discreet. It was performed after antifungal treatment because oral candidiasis could complicate the histologic analysis [13].

The scientific literature has reported several characteristic connections of oral lichen planus with other systemic diseases like diabetes mellitus, hypertension, viral hepatitis (HBV and HCV) thyroid diseases, cardiac diseases, and malignant diseases [14]. Its occurrence in conjunction with autoimmune conditions such as Hashimoto's thyroiditis, rheumatoid arthritis, lupus erythematosus, and Gougerot-Sjogren syndrome has also been reported [15]. However, the association with polycythemia was rarely mentioned. To the best of our knowledge, this clinical report is the second one talking about this possible correlation after the case published by Berbis et al. in 1987 [16] which makes our observation original.

**Figure 6:**
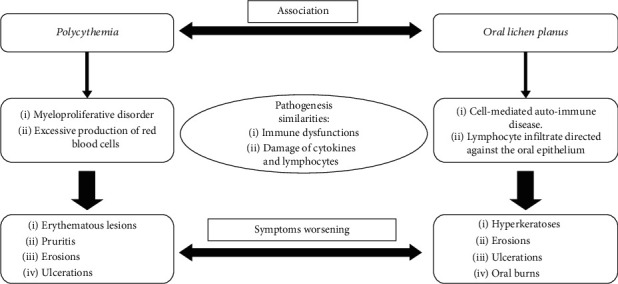
A proposal of an explanation of the association of oral lichen planus and polycythemia.

The coexistence in this case of a myeloproliferative disorder involving red blood cells and possibly damaging the other cell lines and oral lichen planus which is believed to result from an abnormal T-cell mediated immune response directed against the oral epithelium suggests the hypothesis of an association between the two diseases via an immunological link. Gangemi et al. proved in a case-control study that decreased plasma levels of IL-33, a member of the IL-1 cytokine family which is expressed in the nucleus of endothelial cells and epithelial cells of barrier tissues [17], could contribute to the altered function of Th2 lymphocytes in patients with polycythemia [18]. Similarly, Li et al. demonstrated in a mouse model that Th2 lymphocytes' migration to bone marrow under high-altitude hypoxia promotes erythropoiesis via activin A and IL-9 [19]. Thus, we believe that these findings are a concrete reflection of a dysregulation in the immune status by damaging cytokines (IL-33, IL-1, and IL-9) and altering lymphocytes (Th2) which may play a central role in the onset and development of oral lichen planus and polycythemia; these diseases share the similar immunological effector mechanisms and can worsen one another (Figure 6).

As for the treatment, the cooperation between three different teams (oral medicine, hematology, and cardiology) led to obtain the correct diagnosis and helped the patient get the appropriate care. Hydroxyurea (Hydroxycarbamid) and Zyloric (Allopurinol) helped to control polycythemia and to improve the patient's state. Topical corticosteroids and Chlorhexidine mouthwashes contributed to reduce oral burning and local pruritus. Veillet-Lemay et al. reported that Hydroxyurea is responsible of the appearance of oral pigmentations [20] which was seen in our patient. Allopurinol was reported to cause oral lichenoid reactions [21]. However, its temporary withdrawal (for 2 months) did not help to obtain an improvement in symptoms which rules out the diagnosis an oral lichenoid drug reaction (OLDR).

## 4. Conclusion

This paper reports a possible association between oral lichen planus and secondary polycythemia. Such a hypothesis remains to be demonstrated by further studies aiming to improve our knowledge about the causal mechanisms of these two diseases in order to find the appropriate cure. The presence of oral lesions should alert the clinicians. These are key factors in the process of identifying and early diagnosing these diseases. Thus, complications can be prevented and treatment can be started at an early stage, avoiding further damage.

## Figures and Tables

**Table 1:** Evolution of biological variables before and after treatment.

Variable	Common values	Initial values	After treatment
Red blood cells (RBC) [10^6^/*μ*l]]	4.2 to 5.7	6.30	5
Hemoglobin (Hb) [g/dL]	13	19	16
Hematocrit (Ht) [%]	40 to 52	54.5	48
Erythropoietin (EPO) [mIU/mL]	3.7 to 31.5	109	3.9

**Table 2:** The WHO diagnosis criteria for PV (2016) and the diagnosis retained.

WHO diagnostic criteria for PV (2016)	Our patient	Final diagnosis
(1) Major criteria (a) Hemoglobin:16.5 g/dL in men; hemoglobin: 16.0 g/dL in women Or hematocrit: 49% in men; hematocrit: 48% in women Or increased red cell mass (RCM) (b) BM biopsy showing hypercellularity for age with trilineage growth (panmyelosis) including prominent erythroid, granulocytic, and megakaryocytic proliferation with pleomorphic, mature megakaryocytes (differences in size) (c) Presence of JAK2 (V617F) or JAK2 exon 12 mutation	Hb =19 g/dL and Ht =54.4% BM biopsy not realized because of bleeding risk Absence of JAK2 (V617F) mutation	Polycythemia vera (PV) ruled out Polycythemia secondary to chronic hypoxia retained
(2) Minor criteria (i) Subnormal serum erythropoietin level	Increased serum erythropoietin level (109 mIU/mL)	
Diagnosis of polycythaemia vera (PV) requires meeting either all 3 major criteria or the first 2 major criteria and the minor criterion		

## Data Availability

The data used to support the findings of this paper are included within the article.
